# Primary Care Disease Management for Venous Leg Ulceration in German Healthcare: Results of the Ulcus Cruris Care Pilot Study

**DOI:** 10.3390/healthcare11182521

**Published:** 2023-09-12

**Authors:** Jonas D. Senft, Thomas Fleischhauer, Regina Poß-Doering, Jona Frasch, Manuel Feißt, Sinclair Awounvo, Uwe Müller-Bühl, Attila Altiner, Joachim Szecsenyi, Gunter Laux

**Affiliations:** 1 Department of General Practice and Health Services Research, University Hospital Heidelberg, Im Neuenheimer Feld 130.3, 69120 Heidelberg, Germany; 2 aQua Institute for Applied Quality Promotion and Research in Health Care GmbH, Maschmühlenweg 8-10, 37073 Göttingen, Germany; 3 Institute of Medical Biometry, University Hospital Heidelberg, Im Neuenheimer Feld 130.3, 69120 Heidelberg, Germany

**Keywords:** venous leg ulceration, chronic wounds, compression therapy, disease management, general practice, patient education

## Abstract

Despite proven effectiveness, compression therapy is applied in only 20–40% of patients with venous leg ulceration, leading to avoidable chronification and morbidity. The Ulcus Cruris Care project was established to develop a new disease-management concept comparable to existing programs for chronic diseases to support evidence-based treatment of venous leg ulceration. This prospective controlled study assessed its first implementation. Interventional elements comprised online training for general practitioner practices, software support for case management, and educational materials for patients. A total of 20 practices and 40 patients were enrolled in a 1:1 ratio to the intervention and control group. Guideline-conform compression therapy was applied more frequently in the intervention group (19/20 [95%] vs. 11/19 [58%]; *p* = 0.006). For patients with ulcers existing ≤ 6 months, the healing rate at 12 weeks was 8/11 [73%] (intervention) compared to 4/11 [36%] (control; *p* = 0.087). Patients after intervention had higher scores for self-help and education in the PACIC-5A questionnaire (42.9 ± 41.6 vs. 11.4 ± 28.8; *p* = 0.044). Treatment costs were EUR 1.380 ± 1.347 (intervention) and EUR 2.049 ± 2.748 (control; *p* = 0.342). The results of this study indicate that the Ulcus Cruris Care intervention may lead to a significant improvement in care. Consequently, a broader rollout in German healthcare seems warranted.

## 1. Introduction

With a proportion of up to 70%, venous leg ulcerations (VLU) account for the majority of chronic wounds of the lower leg [1]. State-of-the-art treatment of chronic VLU is complex and comprises various elements. Modern wound management performed by regular wound cleaning, debridement, antisepsis, bioburden control and treatment with modern dressings supports healing by maintaining a moist wound environment and controlling inflammation [2,3,4]. Furthermore, pain control is essential, since mobility and quality of life in affected patients may be severely impaired [5]. Besides the administration of analgesics, in the long term, it can only be achieved by correct VLU treatment. However, alongside local wound management, counteracting the underlying venous disease represents the main pillar of VLU treatment to support wound healing and avoid chronification. Compression treatment assists venous return, reduces venous reflux and promotes wound healing by a reduction in tissue inflammation [6]. However, despite its proven effectiveness (evidence class I) [7,8], compression is only applied in 20–40% of patients with VLU in Germany and other European countries [9,10].

Multiple barriers to the implementation of an evidence-based VLU treatment have been identified. At first, a significant lack of knowledge on the caregivers’ side has been reported [11]. Compression is often not applied correctly regarding choice of the device and its application technique [12,13], each having a significant impact on pressure and efficacy [7,8,14]. Furthermore, although patient education is a key factor for adherence to VLU treatment [15,16], research indicates that patients tend to be uninformed regarding disease and in particular compression therapy [17]. Increasing focus on local wound treatment, time pressure in outpatient care, and a lack of standardised care concepts may be further factors driving inefficient care for affected patients who are at risk of suffering avoidable chronification and considerable impairments to their quality of life [18,19].

The Ulcus Cruris Care (UCC) project was established to address these deficits and to support primary care for patients with VLU. In Germany and other countries worldwide, disease-management programs have been successfully implemented to improve the primary care of chronic diseases [20,21,22]. Training and case management elements are a central part of these interventions used to standardise treatment according to current evidence but also to actively involve non-physician medical workforces into treatment in order to improve access to high-quality care. The UCC intervention is the first approach of a standardised care concept to support the treatment of chronic wounds. It was methodically aligned with disease-management programs to use this implementation pathway in primary care. The multifaceted intervention consists of online training for general practitioners (GPs) and medical assistants (MAs), a case management concept and educational material for patients. Its main aims are to promote a broadscale implementation of a standardised and evidence-based treatment of VLU and to support informed patient participation in the treatment process. All interventional elements were aligned with the needs and expectations of caregivers and patients [23].

Following the conceptualization of the UCC intervention, this pilot study exploratively assessed its first-time implementation in a small-scale setting. Data on indicators of potential interventional effects like the application of guideline-conform compression therapy, wound healing, patient-reported outcomes, adherence, and treatment costs were collected prospectively for explorative analysis and to serve as a basis for further confirmative studies.

## 2. Materials and Methods

### 2.1. Study Design

**Figure 1:**
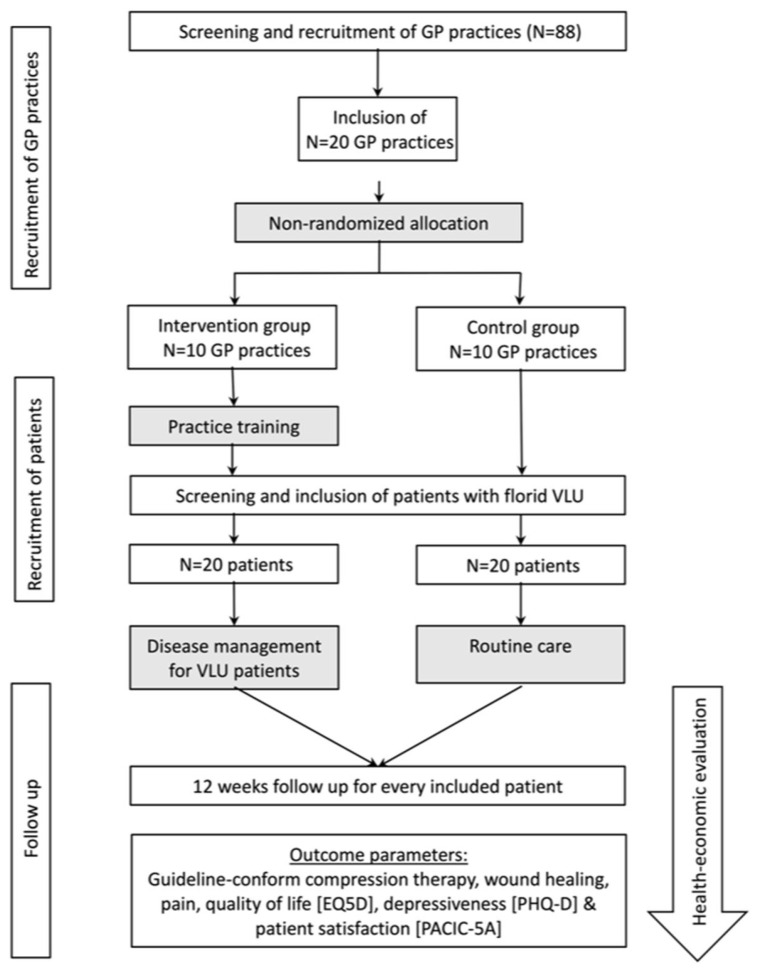
Flow chart of the Ulcus Cruris Care pilot study.

A multi-centre, non-randomised controlled study was conducted in 20 GP practices from 1 October 2021 until 31 January 2022. The UCC intervention was implemented in 10 GP practices in the intervention group (IG), while 10 GP practices served as the control group (CG). Practices were allowed to recruit study patients until the recruitment target of 20 cases was reached in each study arm (Figure 1). Outcome parameters were collected prospectively for 3 months. The study design was approved by the Ethics Committee of the Medical Faculty, University Heidelberg (Nr. S-416/2020). The study protocol was registered in the German Registry of Clinical Trials (https://drks-neu.uniklinik-freiburg.de/drks_web/) accessed on 19 February 2021, under registration number DRKS00024421.

### 2.2. Inclusion and Exclusion Criteria

Study practices were recruited in the German federal states Baden-Wuerttemberg, Hesse and Rhineland-Palatinate, and were eligible for inclusion if they had at least one certified Health Care Assistant (German: “Versorgungsassistent/in in der Hausarztpraxis”, VERAH) employed and had at least one internet-enabled computer ready for the installation of the case management environment “CareCockpit” [24]. In Germany MAs may undergo an additional training of 200 hours to become a Health Care Assistant and usually have a wider area of responsibilities, such as case management activities, home visits and wound management [25]. Practices were included after informed consent was obtained from participating GPs and Health Care Assistants. Since intervention elements were under development at the study start, the first 10 GP practices were included as a control; thereafter, the intervention was implemented in the following 10 GP practices. 

Patients were screened and included upon informed consent by participating GPs. Patients with VLU were eligible for inclusion if venous disease was considered as the leading aetiology of the ulceration (Table 1). Measurement of the ankle–brachial pressure index or duplex sonography were not required to be routinely performed upon patient inclusion in order to avoid unintended effects regarding diagnostic pathways of routine care within the control group, since diagnostics for VLU were also part of training within the UCC intervention. 

Patient screening and inclusion were stopped by the study centre once the recruitment target of 20 patients per study arm had been reached. Patients could withdraw participation any time. Participating GP practices were instructed to report drop-out patients or loss-of-follow-up to the study centre.

### 2.3. Intervention

The UCC disease management intervention aims to foster standardised evidence-based and patient-centred VLU treatment in GP practices using a three-module concept:

a. Online team training for GPs and Health Care Assistants
b. Software support for case management
c. Educational material and e-learning for patients

Online team training was performed via a live online webinar and a target group specific e-learning course using the Articulate360 platform (Articulate Global, LLC, New York, NY, USA) with an approximate duration of 60 minutes each. The live online webinar was held by experts from the department of General Practice and Health Services Research at the University Hospital Heidelberg. Webinar and e-learning courses were based on standardised teaching blueprints with learning targets and were interdisciplinarily reviewed by experts from the fields of phlebology, dermatology, and vascular surgery. Educational contents comprised diagnostics, compression therapy, local wound treatment, indications for specialist referral and patient education based on current evidence. The e-learning courses provided additional illustrations and supportive digital media, such as a video on the standardised application of compression bandages, relevant practical guides like the recognition of bacterial contamination, measurement of the ankle brachial pressure index, and overviews of wound dressings and their properties. Practices were allowed and encouraged to share digital media, for instance with care services involved in the treatment. 

A software module within the CareCockpit [24] case management environment was designed to guide through a standardised wound visit. As a central part of online team training, MAs and GPs were taught to routinely perform comprehensive wound care consisting of four main visit elements: patient education, compression therapy, wound assessment, and wound management. MAs were trained to assess and monitor patients with VLU using the CareCockpit tool as a guide during wound visits. Main features comprised standardised on-click documentation with overviews of the course of disease and the assisted creation of a treatment plan. Furthermore, standard operating procedures (SOPs) summarising guideline-conform recommendations were provided within the case management environment to support standardised evidence-based treatment. SOPs for compression treatment comprised the choice of an agent according to the situation (decongestion phase or after decongestion) and instructions regarding practical application [26]. Recommendations for wound cleansing comprised the choice of method and its practical exertion [4]. The first-line wound-cleansing method was mechanical cleansing by a sterile compress and irrigation using saline solution. In cases of clinical signs of bacterial contamination, local antiseptic treatment by appropriate agents like octenidine or polyhexanide was recommended, and rules regarding its practical application regarding exposure time were illustrated. Fibrin-coated wounds were recommended to be cleaned by small surgical debridement or use of enzymatic agents. Instructions also comprised a phase-appropriate choice of wound dressings [3]. However, all SOPs were intended to serve as orientation regarding evidence-based VLU treatment, and adherence was not mandatory. All GP practices were free and independent in their choice of treatment. 

Educational materials for patients were conceived by the department of General Practice and Health Services Research and reviewed by a local association for patient support and self-help (Gesundheitstreffpunkt Mannheim, Mannheim, Germany). Patient information sheets outlined basic information about the disease, diagnostics, and treatment as well as appropriate general measures such as physical activity, skin care, and handling of compression devices using common language and illustrations. Printout versions of the information sheets were accessible for MAs using the CareCockpit tool. Furthermore, based on the information sheets, an additional e-learning course for patients was conceived as a digital alternative. All educational materials were conceived with the intention of serving as an addition to regular oral patient information in GP practices. MAs and GPs were outlined basic information about the disease, diagnostics, and treatment as well as appropriate general measures such as physical activity, skin care, and handling of the compression devices

No intervention was performed in practices included in the control group.

### 2.4. Outcome Parameters

Data referring to outcome parameters were collected prospectively by participating GP practices at baseline (T0), after 4 weeks (T1) and after 12 weeks (T2) in an electronic case report form and transferred in a pseudonymised form to the study centre for analysis.

Guideline-conform compression treatment for VLU was considered fulfilled if devices applied during the decongestion phase were suitable for exerting an adequate working pressure of ≥40 mmHg, corresponding to compression class III [26]. After wound healing, the application of medical compression stockings was required. 

Wound healing was defined as complete epithelialization of the wound without scab and was determined by the physicians of the participating GP practices at T2. Wound area was approximated by measuring the greatest length and greatest width in cm according to the perpendicular method and approximating the corresponding oval area size in cm^2^. Patient-reported outcomes comprised quality of life (EQ-5D-5L) [27], pain measured by a visual analogue scale (VAS) and depressiveness (PHQ-D) [28]. The Patient Assessment of Chronic Illness Care (PACIC-5A) [29] was used to survey patient satisfaction with care and patient education. 

Adherence was assessed by participating Health Care Assistants estimating the degree of patient adherence to the treatment on a scale of 0–100%. An adherence rate of >80% was classified as adherent, 20–80% as partially adherent, and <20% as non-adherent.

Health economic analysis comprised assessment of costs for VLU treatment and cost-effectiveness of the intervention. Relevant health care cost data were obtained from documented prescriptions at T2. Outpatient and inpatient contacts, medical aids, and remedies were valued using standard unit costs [30]. Prescription drugs and wound dressings were valued with pharmacy retail prices when available and with pharmacy purchase prices otherwise, both obtained from the IFAP database [31]. Care services recorded encompassed ambulatory care and household help. 

### 2.5. Statistical Analysis

Statistical analysis was performed using R software version 4.2.1. Patients’ demographics and study outcomes were assessed descriptively in both study groups. Continuous outcome variables were described using mean ± standard deviation. For categorical variables, absolute and relative frequencies were provided. The intervention and the control group were compared using the Mann–Whitney U test for continuous variables and the Chi-square test for frequencies. All statistical tests were two-sided and conducted at a 5% significance level. In addition, 95% confidence intervals were also reported. For the health economic evaluation, cost effectiveness was analysed using an incremental cost effectiveness ratio (ICER): ICER = $\frac{{costs}_{intervention}-{costs}_{control}}{{effect}_{intervention}-{effects}_{control}}$. Changes in health-related quality of life (HR QoL) during the observation period as well as the rate of wound healing served as effect measures. Due to the explorative nature of the study, no formal sample size calculation was performed and *p*-values have to be interpreted in a descriptive sense.

## 3. Results

### 3.1. Patient Recruitment and Baseline Characteristics

Participating GP practices recruited patients between 1 December 2020 and 27 October 2021 when the target sample power of 20 patients was reached in both study arms. Within CG, 20 patients were recruited by seven practices: two practices recruited 6 patients each, one practice included 3 patients, one practice included 2 patients and three practices included 1 patient. Within IG, 20 patients were included by nine study practices: two practices recruited 4 patients each, two practices included 3 patients each, one practice included 2 patients and four practices included 1 patient. One patient in the CG deceased after T1 with no causal relation to the intervention. There were no dropouts and follow-up data were obtained for all included patients. Subjectively assessed health status with the EQ-5D-5L showed higher values in the CG at T0. No further differences were found between the IG and CG regarding baseline characteristics (Table 2).

### 3.2. Use of Guideline-Conform Compression Therapy

Guideline-conform compression therapy was applied more frequently in the IG (19/20 [95%] IG vs. 11/19 [58%] CG; *p* = 0.006). All patients in the IG received compression; in the CG 3 of 19 [16%] patients did not receive compression at all [*p* = 0.064]. Compression agents applied during the decongestion phase comprised 2-layer bandages (IG: 16/20 [80%], CG: 9/19 [47%]), multi-component bandages (IG: 1/20 [5%]), ulcer stocking systems (IG: 2/20 [10%], CG: 1/19 [5%]), medical adaptive compression systems (CG: 1/19 [5%]) and medical compression stockings (IG: 1/20 [5%], CG: 5/19 [26%].

Within the IG, 9 of 10 patients [90%] received medical compression stockings for recurrence prophylaxis after the wound was healed, compared to 1 of 7 [14%] in the CG [*p* = 0.002].

### 3.3. Wound Healing

For patients with VLU with a duration ≤ 6 months at baseline, healing rate at 12 weeks was 8/11 [73%] (IG) compared to 4/11 [36%] (CG) (*p* = 0.087). Overall healing rate at 12 weeks was 10/20 [50%] (IG) vs. 7/19 [37%] (CG) (*p* = 0.408). The change in wound size at 12 weeks was −6.3 ± 38.8 cm^2^ (IG) and −0.2 ± 14.3 cm^2^ (CG), respectively (*p* = 0.722). One patient in the IG had a recurrence, while no relapses were reported in the CG (*p* = 0.323). 

### 3.4. Patient-Reported Outcomes and Adherence

For the patient-reported outcomes pain, quality of life and depressiveness, there were no notable differences between both groups at 12 weeks (Table 3). No difference was found regarding overall patient satisfaction at T2 according to PACIC-5A (67.3 ± 24.5 IG vs. 68.1 ± 18.5 CG; *p* = 0.872). Patients of the IG had significantly higher scores for the domain “Self-help and education” within the PACIC-5A (42.9 ± 41.6 IG vs. 11.4 ± 28.8 CG; *p* = 0.044). The rate of patients who were considered adherent to treatment did not differ between the groups (18/20 [90%] IG vs. 18/20 CG [90%]; *p* = 1). 

### 3.5. Health Economic Analysis

Treatment costs for VLUs and its secondary effects accumulated to EUR 1.380 ± EUR 1.347 in the IG and EUR 2.049 ± EUR 2.748 in the CG. No inpatient costs occurred. The corresponding ICER was $\frac{-669\;€}{-0.05}$ for HR QoL and $\frac{-669\;€}{13\%}$ for the rate of wound healing. Table 4 shows the treatment costs in both groups.

## 4. Discussion

This study accompanied the first implementation of a disease management intervention for chronic wounds in German primary health care. The results show that the participation of GP practices and patients in the UCC intervention leads to a higher use of guideline-compliant compression for VLU treatment. Though a consecutive beneficial effect on wound healing and patient education may be assumed, no firm conclusion can be drawn in this regard.

In venous ulceration, healing is impaired by underlying disease requiring causal treatment. Compression therapy counteracts venous hypertension and leads to faster healing [7]. Compared to routine care, analyses showing the application of guideline-conform compression treatment in 20–40% of VLU patients [9,10], a rate of 100% in the intervention group indicates a high effectiveness of the UCC intervention in this regard. Although compression is a main pillar of evidence-based VLU treatment, it is not regularly applied due to multifactorial reasons. First, studies have demonstrated a knowledge deficit among care providers [12,13]. On the other hand, there is a broad complexity in the routine care of chronic wounds with many participating actors, such as hospitals, specialised nursing services and certified wound managers focusing on modern local wound treatment. Patients tend to assume a rather passive role and are not well informed about the evident efficacy of compression [17]. Treatment may be associated with discomfort and in contrast to obvious treatment strategies like cancer surgery, it is not intuitively clear to patients that compression promotes wound healing. Understanding its efficacy, working mechanism and appropriate use is fundamental for informed patient participation. Hence, educational interventions may be a key factor for adherence [15,16]. The UCC intervention addresses these deficits in a multifaceted manner by promoting knowledge among caregivers as well as patients and supporting standardised guideline-conform treatment by case management. With regard to surgical treatment, training also emphasised the effectiveness of causal interventional approaches, particularly in patients with significant superficial venous reflux [32]. None of the included patients received venous ablation, probably due to the reality of outpatient care in Germany, which currently has no routine pathway for VLU patients to receive interventional treatment. While this deficit has to be further addressed, it cannot be solved solely on the level of GP care, but rather by the close networking of GPs, phlebologists and specialists experienced in endovenous ablation.

The rate of healed ulcers that had existed ≤ 6 months at T0 was substantially higher in the IG (73%) than in the CG (36%). Thus, the results strongly suggest an effect of the UCC intervention on would healing. This assumption is supported by the fact that the appropriate use of compression therapy is proven to lead to faster healing [7,26]. The low healing rates in the CG might be due to a lack of treatment of the underlying venous disease. This major deficit in routine care was a driving incentive for the UCC project and development of the described intervention. Today, modern wound care strongly focusses on wound management and the choice of applied dressings, while causally orientated treatment seems to be more and more neglected. Treatment of the underlying disease needs to be in the centre focus of wound treatment, regularly monitored, and its barriers have to be identified and addressed. The UCC intervention trains MAs and GPs not only to use compression therapy, but also to continuously assess causal treatment and, if necessary, to interfere by engaging in educational measures to address adherence or by communicating with specialist physicians and nursing services in case of inadequate treatment. 

The results of this study, particularly regarding the domain “self-help/education” within the PACIC-5A, indicate an effect regarding empowerment for self-care, which was another main goal of the intervention. Active involvement and patient education support wound healing according to grade 1A evidence and should be a pillar of VLU treatment [16]. Patient education supports wound healing according to grade 1A evidence and, therefore, should be a main component of VLU treatment. Participating MAs and GPs were trained to routinely assess and promote patient education to move patients from a passive role during wound dressing change to an active participation in wound treatment. They addressed important issues for self-care, such as body and skin care and the handling of compression devices, as well as recommendations for movement exercises, which have been shown to promote wound healing in VLU [33]. Furthermore, GP practices were encouraged to actively involve patients and relatives in regular dressing changes at home. The results of this study indicate that these efforts support participation, which may be a key factor for patient adherence to VLU treatment. 

With regard to health care costs, the analysis showed a mean cost saving of EUR 669per 3 months of treatment for practices following the UCC intervention. However, the sample size did not yield a power high enough to demonstrate a statistically significant effect. Cost saving was mainly attributable to lower mean costs for wound dressing materials and therapeutics per patient. Training within the UCC intervention aimed to raise awareness of the economic use of wound dressings. It may also be assumed that faster wound healing in the IG may result in lower GP prescription costs. With regard to cost effectiveness, the ICER analysis suggests that the UCC intervention may lead to cost savings coupled with improvements in wound healing on the one hand, yet smaller improvements in the HR QoL over a period of three months. However, it seems probable that differences in HR QoL changes are rather random and due to low sample size.

## 5. Strengths and Limitations

To our knowledge, this is the first study assessing a comprehensive disease-management approach for VLU patients in primary care. The limitations of this study are mainly represented by its small sample size and its explorative design. Planned as a pilot study subsequent to conceptualization of the UCC intervention, the small-scale setting was chosen for the first-time implementation and explorative analysis of potential interventional effects. Consequently, firm conclusions regarding effects on wound healing, HR QoL, or cost-effectiveness cannot be drawn at this point. Furthermore, random differences between IG and CG have to be considered, and unwitting selection bias cannot fully be excluded due to the non-randomised setting. However, the results of this study indicate an improvement in care for VLU patients and serve as a basis for a broader roll-out of the UCC intervention, which will be assessed by a subsequent C-RCT.

## 6. Conclusions

This non-randomised prospective cohort study evaluated the first implementation of a disease management intervention for VLU in German primary care. Findings indicate that the participation of GP practices and patients in the UCC intervention may lead to a significant improvement in care, particularly with regard to a higher use of guideline-conform compression therapy. Consequently, a broader rollout of this disease-management concept in a subsequent RCT seems warranted and may pave the way for a countrywide disease-management program. In this way, routine care for patients affected by VLU may be improved on a large scale, and the UCC intervention could serve as a model for other countries.

## Figures and Tables

**Table 1:** Inclusion and exclusion criteria for patients.

Inclusion Criteria	Exclusion Criteria
Physician-diagnosed, confirmed VLU	Peripheral arterial occlusive disease of affected lower extremity with ABPI < 0.5 or ancle artery pressure < 50 mmHg
Age ≥ 18 years	Age < 18 years
	Pyoderma gangraenosum
	Diabetic foot syndrome
	No capacity to consent

VLU = venous leg ulceration, ABPI = ankle-brachial pressure index.

**Table 2:** Baseline characteristics of patients at T0.

	Intervention (n = 20)	Control (n = 20)	Total (n = 40)	*p*-Value *
Male (%)	7 (35%)	10 (50%)	17 (42%)	0.337
Age				
Median (IQR)	76.5 (62.0–86.0)	77.5 (69.5–82.5)	77.0 (67.5–84.0)	0.807
Ulcer duration [months]				
Median (IQR)	4.5 (1.0–8.5)	4.5 (1.5–24.0)	4.5 (1.0–9.0)	0.275
Charlson Comorbidity Index				
Median (IQR)	4.0 (2.5–5.0)	3.0 (2.5–4.0)	4.0 (2.5–5.0)	0.423
Wound size [cm^2^]				
Median (IQR)	3.5 (1.0–7.0)	1.0 (1.0–10.0)	2.5 (1.0–7.5)	0.804
Pain (VAS)				
Median (IQR)	3.0 (0.5–5.0)	4.0 (2.0–6.0)	3.0 (1.5–5.5)	0.167
Depressiveness (PHQ-9)				
Median (IQR)	4.5 (2.5–6.0)	5.0 (1.5–7.0)	5.0 (2.0–6.0)	0.892
Quality of life (EQ-5D-5L)				
Median (IQR)	60.0 (47.5–72.5)	72.5 (55.0–95.5)	67.5 (50.0–82.5)	0.043 *

Continuous values are reported as median (interquartile range) and IQR = Interquartile range. * Mann–Whitney U test was used for comparison between intervention and control group.

**Table 3:** Summary of endpoints collected at 12 weeks after inclusion (T2).

Outcome	Intervention (T2) n = 20	Control (T2) n = 19	*p*-Value *	95%-CI
Pain (VAS)	1.79 ± 2.15	1.74 ± 1.85	0.928	[−1, 1]
Δ T2-Baseline	−1.21 ± 2.95	−2.11 ± 2.56	0.393	[−1, 2]
Depressiveness (PHQ-9)	4.8 ± 5.2	3.5 ± 4.1	0.186	[−1, 4]
Δ T2-Baseline	−0.85 ± 3.6	−0.94 ± 2.4	0.652	[−1, 2]
QoL score (EQ-5D-5L)	70.3 ± 24.3	80.7 ± 21.4	0.166	[−28, 2]
Δ T2-Baseline	12.5 ± 18.3	9.7 ± 21.5	0.364	[−5, 12]
QoL Index (EQ-5D-5L)	0.71 ± 0.36	0.83 ± 0.26	0.297	[−0.21, 0.053]
Δ T2-Baseline	0.05 ± 0.19	0.11 ± 0.16	0.817	[−0.12, 0.083]

Values are reported as mean ± standard deviation. Δ Delta = Difference between value at T2 (12 weeks) and T0 (Baseline). * Mann–Whitney U test was used for comparison between intervention and control group.

**Table 4:** Venous leg ulcer treatment costs.

Costs	Intervention	Control	*p*-Value *
Outpatient care	EUR 172 ± 76	EUR 155 ± 176	0.693
Specialist care	EUR 7 ± 33	EUR 5 ± 22	0.780
GP care	EUR 164 ± 71	EUR 142 ± 134	0.522
Care services	EUR 498 ± 888	EUR 378 ± 658	0.630
Remedies	EUR 13 ± 34	EUR 10 ± 28	0.804
GP Prescriptions	EUR 697 ± 1.029	EUR 1.506 ± 2.597	0.207
Medical aids	EUR 223 ± 454	EUR 647 ± 1.575	0.260
Wound dressings	EUR 276 ± 348	EUR 625 ± 1.014	0.159
Total costs	EUR 1.380 ± 1347	EUR 2.049 ± 2.784	0.342

Values are reported as mean ± standard deviation, GP = general practitioner. * Mann–Whitney U test was used for comparison between intervention and control group.

## Data Availability

The datasets analysed during the current study are available from the corresponding author on reasonable request.

## Abbreviations

CG	Control group
CI	Confidence interval
GP	general practitioner
HR QoL	health-related quality of life
IG	Intervention group
IQR	Interquartile range
UCC	Ulcus Cruris Care
VAS	visual analogue scale
VLU	venous leg ulceration
